# Mental but no bio-physiological long-term habituation to repeated social stress: A study on soldiers and the influence of mission abroad

**DOI:** 10.3389/fpsyt.2022.1011181

**Published:** 2022-12-15

**Authors:** Tanja Maier, Manuela Rappel, Dae-Sup Rhee, Sebastian Brill, Julia Maderner, Friederike Pijahn, Harald Gündel, Peter Radermacher, Benedikt Friemert, Horst-Peter Becker, Christiane Waller

**Affiliations:** ^1^Clinic for Psychosomatics and Psychotherapeutic Medicine, Ulm University Medical Center, Ulm, Germany; ^2^Military Hospital, Ulm, Germany; ^3^Institute for Anesthesiological Pathophysiology and Process Engineering, Ulm University, Ulm, Germany; ^4^Military Hospital, Berlin, Germany; ^5^Department of Psychosomatics and Psychotherapeutic Medicine, Paracelsus Medical University, Nuremberg, Germany

**Keywords:** social stress, mission abroad, TSST-G, habituation, biological stress axis response, salivary cortisol, salivary α-amylase, heart rate variability

## Abstract

Soldiers regularly participate in missions abroad and subjectively adapt to this situation. However, they have an increased lifetime cardiovascular risk compared to other occupational groups. To test the hypothesis that foreign deployment results in different stress habituation patterns, we investigated long-term psychological and bio-physiological stress responses to a repeated social stress task in healthy soldiers with and without foreign deployment. Ninety-one female and male soldiers from the BEST study (German armed forces deployment and stress) participated three times in the Trier Social Stress Test for groups (TSST-G) prior to, 6–8 weeks after and 1 year after the mission abroad and were compared to a control group without foreign deployment during the study period. They completed the State-Trait-Anxiety Inventory scale (STAI), the Primary Appraisal Secondary Appraisal questionnaire (PASA) and the Multidimensional Mood State Questionnaire (MDBF). Salivary cortisol and α-amylase, blood pressure, heart rate and heart rate variability were determined. Soldiers showed mental habituation over the three times with a significant decrease after the TSST-G in anxiousness (STAI) and cognitive stress appraisal (PASA), they were calmer and reported better mood (MDBF). Prior to the social stress part, the mood (MDBF) declined significantly. None of the biological and physiological markers showed any adaptation to the TSST-G. Mission abroad did not significantly influence any measured psychobiological marker when compared to soldiers without foreign deployment. Foreign deployment does not result in alterations in psychobiological social stress response patterns over 1 year after mission abroad which indicates that adaptation to acute social stress is highly maintained in healthy soldiers. The discrepancy between subjective perception and objective stress response has numerous clinical implications and should receive more attention.

## 1 Introduction

The importance of social stress in the general public awareness has strongly increased over the years and inspired research in different kind of fields (1). Chronic social stress is considered as a psychobiological risk factor (2) for cardiovascular diseases (CVD) (3), e.g., myocardial infarction or high blood pressure (4); moreover it shows robust causal associations with depression and anxiety (5, 6). Experiencing stress results from individual factors, e.g., lack of social support, time pressure, social conflicts, threats or hazardous situations (7–9).

Some professions are facing greater stress due to their job task, e.g., military personnel who are often exposed to various stressors like heavy workload, hierarchical structures or hazardous experiences during combat which may all lead to high stress perception (10). Not all missions abroad result in traumatic stress or experiencing direct mortal danger but soldiers are mostly facing social stress. Soldiers show higher risk for perceived job-stress than civilians and high rates of cardiovascular disease, particularly during foreign deployment (11). Soldiers with such missions abroad are also more likely to suffer from hypertension because of increased mental stress than non-deployed soldiers (12, 13). It is known that almost half of the deployed soldiers experience at least one traumatic event and hence have a two-to fourfold elevated risk of developing mental disorders like PTSD (14). This raises the question if soldiers that undergo missions abroad would react differently to social stress afterwards.

Various stress models demonstrated that the extent whether an external stressor is perceived as stressful or not depends on the individual coping mechanism, social support and resources (15). Especially psychosocial stress factors are closely linked to physical and mental wellbeing (16). Anxiety with the feeling of tension is the leading emotion linked to the stress response system which has an impact on cognition, emotions and behavior through the activation of the autonomic nervous response system (ANS) (16). The human body initiates elaborate reactions to stress and shows different coping mechanism to maintain homeostasis. Social stress activates the neurohumoral response of the hypothalamic-pituitary-adrenal (HPA) axis and the sympathetic-adrenal-medullary (SAM) system (17) which both modulate and show a bidirectional interaction with the immune system (18). The HPA axis releases cortisol which stimulates the release of fatty acids for providing energy and interacts through hormones with other systems (e.g., nervous, metabolic) (19). In contrast the SAM system provides epinephrine and norepinephrine to prepare for a fight or flight reaction (e.g., increase in heart rate and blood pressure) (20). Ongoing stress over an extended time period can lead to chronic modulation of the stress response system and modified gene expression which can cause dysregulated immune function (18).

However, recent studies have also focused on the adaptation process to a repetitive stressor (21) which is called “habituation.” This term is generally referred to as a reduction of the measured variable due to a repeated stimulation (22) and is independent of the stimulus and the extent of habituation. Thompson and Spencer tried to define criteria for a more operational approach for habituation, which composes of e.g., the influence of frequency or strength of the stimulus as well as the phenomenon of spontaneous recovery or potentiation of habituation (22). It has been frequently shown that the HPA-axis response habituated to repeated psychosocial stress stimuli both in experimental animals (23, 24) as well as in human studies (21). However, the mechanism and stability of this phenomenon is not completely understood. Some research indicated that the attenuated HPA-axis response is a mixture between negative feedback mechanisms induced by cortisol and learning/memory and can only partially be explained by habituation (25). This resulted in the concept to differentiate between high and low responders (26). Results for the SAM axis are inconsistent with comparatively lower habituation compared to the HPA-axis, e.g., heart rate decreases whereas epinephrine and norepinephrine release showed no habituation (27). Mental habituation to repeated social stress can be measured by quantifying behavioral changes (28). Subjective perception, estimations of the situation like tenseness, irritation or the feeling of stress significantly decrease over time (28). Monkeys showed a decrease in behavioral stress reaction whereas no biological habituation (decrease in cortisol levels) was detected (29). These findings indicate that changes in mood or behavior may not necessarily reflect underlying biological or physiological processes in response to repeated stress (30).

To properly examine changes in the biological, physiological and psychological stress response a valid and repeatable stress exposure protocol and considerations about the stress parameters are required. The Trier Social Stress Test for Groups [TSST-G (31)] is suitable for this purpose and is therefore used as a standard tool in psychobiological research. The applicability for inducing multidimensional stress in participants (HPA-axis response, SAM system activation and subjective emotional stress) has been frequently tested (32, 33). The TSST can be used for measuring repeated stress, whereas the protocol is often slightly adapted over time. To capture HPA-axis response and habituation the determination of salivary cortisol is standard practice whereas the SAM-system can be measured through salivary α-amylase (sAA) activity or adrenaline/noradrenaline concentrations (34, 35). Cardiovascular effects that indirectly mirror SAM activity can be revealed through heart rate variability (HRV) and blood pressure. Subjective psychological activation and differences over time include appraisal, anxiety, alertness, and mood (35). Recent studies indicate a habituation effect of cortisol to a repeated stressor whereas no decrease or even a slight increase over time has been described for sAA and HRV (21, 27, 36). Blunted cortisol concentrations have been described for deployed soldiers who experienced high stress (development of PTSD) in comparison to civilians or less stressed soldiers as measured with the Trier Social Stress Test (TSST) (37, 38).

To test the hypothesis that foreign deployment results in attenuated habituation patterns, we measured standardized psychological, physiological and biological markers in a TSST-group setting with a 1 year distance for the follow-up measure. Of further importance was the assessment of differences in the psychological and biological stress effects of the repeated mental stress tasks in healthy soldiers. The study is part of the BEST study (German Armed Forces Deployment and Stress).

## 2 Materials and methods

### 2.1 Recruitment and study design

**FIGURE 1:**
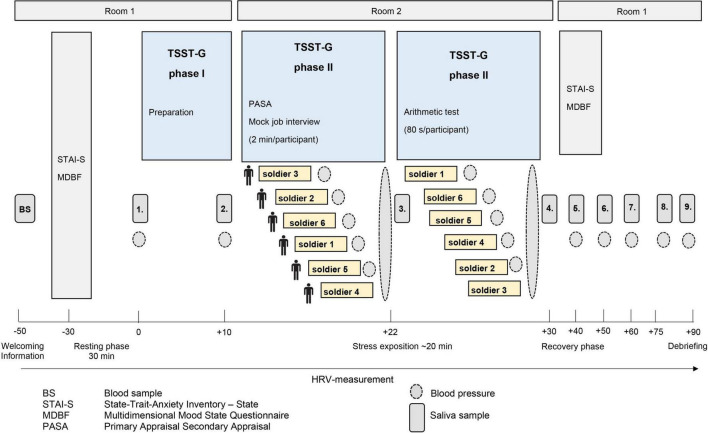
Illustration of the standardized process description of the trier social stress test for groups (TSST-G). The trial design was adapted according to von Dawans, Kirschbaum and Heinrichs (31).

The participants from the BEST-study were German soldiers that had been recruited from military barracks in Dornstadt, Laupheim, and Ulm as well as in the German Armed Forces hospital in Ulm. The aim of the prospective study design was to investigate biopsychosocial stress effects of foreign deployment on cardiovascular health in the German Armed Forces. Recruited soldiers with similar age and gender distribution without mission abroad served as the control group. All participants repeatedly passed through a social stress task, the TSST-G (31) at three different time points, prior to (t0), and 4–6 weeks (t1) as well as 1 year (t2) after mission abroad. The TSST is well-known and the protocol is widely used for various stress paradigms because it effectively leads to psychobiological stress response and has high reliability (31). All sessions took place and were scheduled at the same time in the early evenings and participants were asked not to eat or drink anything else than water at least 1 h beforehand. Each TSST-G lasted about 3 h in total, depending on the number of participants (3–6 per group). The standardized protocol (Figure 1) consisted of a 30 min resting period, followed by a stress exposition lasting for 30–40 min comprising of a 5 min mock job interview (describing their own personality traits) and a mental arithmetic task in front of two specialized evaluators in lab coats, a video camera and in the presence of the other participants. This procedure ensures stress induction not only during the tasks themselves but also through attending the other participants’ tasks. After the stress exposure, a resting period of 60 min was allowed for downregulation of the neuroendocrine systems. Soldiers from the control and experimental group were tested under the same conditions. Various stress-sensitive psychological and biological values were measured at the three time-points for baseline and during stress exposure. The number and time frame of measurements within the TSST-G differed for each variable (Figure 1). Due to the time-sensitive characteristics of the examined variables the study protocol was thoroughly followed and group size did not influence measurement timing. Anamnestic, sociodemographic and psychological information was collected through questionnaires.

### 2.2 Participants

A total of 234 soldiers with regular medical examinations were included and completed t0. Due to administrative changes at the German Armed Forces, the survey of questionnaires had to be paused for 6 months so that 42 participants had to be excluded from final analyses due to missing questionnaires. A total of 91 soldiers completed all three measurements. The biological variables cortisol, amylase, HRV, systolic and diastolic pressure were available with varied quantities and case numbers because of one or more of the following reasons: errors within the technical devices, too little sample material for the analyses, transmission errors and conservative calculations for the changes over time where missing values were excluded and not extrapolated. Forty-four soldiers had a mission abroad between t0 and t1 during the study whereas 47 soldiers were in the control group. The experimental group was mostly deployed to Mali, Afghanistan and the Republic of Kosovo. The mission orders mainly included to build a “Safe and Secure Environment,” securing peace and human security as well as humanitarian aid and training missions.

### 2.3 Biological measures

During the TSST-G nine saliva samples were taken (Figure 1), immediately centrifuged at 3,000 rpm for 5 min, frozen and stored at −80°C until analysis. Cortisol concentrations (nmol/l) were measured using a chemiluminescence immunoassay with high sensitivity (IBL International, Hamburg, Germany). The intra- and interassay coefficients were below 8%. SAA concentrations (U/ml) were analyzed using an enzyme kinetic method and interference measurement was obtained at a wavelength of 405 nm using a standard ELISA reader. Increases in absorbance were calculated for unknowns and standards.

### 2.4 Physiological measures

Heart rate variability and heart rate was derived using the eMotion Faros 180° biosensor (BlindSight GmbH, Schlitz, Germany). All participants were monitored using a three-lead digital electrocardiogram with a sampling rate of 500 Hz during the whole procedure. At 10 time points of the TSST-G 40 s tracings of the HRV were used whereas the number of these 40 s segments varied between the time points. The shortest included interval was during the mental arithmetic task, the longest one during preparation phase (see Figure 1). Data were edited using the HRV Analysis Software (MindWareTechnologies; Version 3.1.7.). Artifacts were identified and manually removed and arrhythmic beats could be averaged with the midbeast function to avoid distorted values. Through time domain measures the standard deviation of all normal-to-normal inter-beat intervals (SDNN) and root mean square of successive differences between normal-to-normal inter-beat intervals (RMSSD) were calculated (39). Diastolic and systolic pressure (mm/Hg) were measured nine times during the TSST-G using the Boso Medicus Control Blood Pressure Monitor (Bosch & Sohn GmbH u. Co. KG, Jungingen, Germany). Soldiers had been instructed how to use the upper arm cuff and started the measuring by themselves after a signal. All values were stored automatically by the devices.

### 2.5 Psychological measures

Acute anxiety was measured using the State-Trait-Anxiety Inventory—State [STAI-S (40)] scale right before and directly after the stress task. “I am tense,” “I am worried,” “I feel calm” are typical phrases for the current emotional state in the STAI-S. Reverse coded items were inverted prior to the analysis. The total sum score of all items was used, higher values indicated more severe anxiety. The Primary Appraisal Secondary Appraisal [PASA (41)] questionnaire covered situational anticipatory cognitive appraisal with statements like “The situation is important for me,” “I do not feel threatened by the situation” before undergoing the stress task. The overall stress index derived from the PASA questionnaire is a difference calculation of a combination of the four primary scales challenge, threat, control expectancy and self-concept of own competencies. Reverse coded items were inverted prior to the analysis and higher outcomes indicated more pronounced stress-appraisal. Soldiers’ mood before and after the stress task was quantified with the Multidimensional Mood State Questionnaire [MDBF (42)]. It is designed for follow-up measurement and consists of two parallel halves. The three bipolar dimensions are good/bad mood, alertness/tiredness and calmness/restlessness including questions like “Right now I feel rested,” “Right now I feel alert.” Negative items were inverted and item sum scores of the three bipolar dimensions were calculated and once again higher outcomes indicated better mood. All instruments are validated and widely-used in connection with the TSST-G and stress research (43–45).

### 2.6 Data analysis

For all biological and physiological data, the changes over the time period during the TSST-G were considered because the calculation of mean differences or using only one timepoint of measurement does not provide sufficient information. Therefore, the area under the curve (AUC) was used. It is an index reflecting the area under the concentration-time curve and generated over all measures of e.g., salivary cortisol during the TSST-G (46). Pruessner et al. (47) showed that this proceeding leads to compressed data for statistical analyses. To examine the sensitivity of the system and changes over time, the area under the curve with respect to increase (AUCi) was calculated. Negative outcome was possible due to mathematical characteristics of the calculations. To examine the overall intensity of the stress response the area under the curve with respect to the ground (AUCg) was calculated. Calculations, editing and graphics were realized using IBM SPSS Statistics 23 (48). Descriptive analyses, *t*-Test for baseline differences between female and male soldiers and Shapiro Wilk tests for item distribution were performed. Shapiro-Wilk test for normal distribution for mostly all stress parameters was significant (*p* < .001). Research showed that ANOVAs are robust against violation of the normal distribution (49). Furthermore, according to the central limit theorem (law of large numbers) the given sampling distribution was approximately normally distributed (50) because the sample size was *N* ≥ 30 for each variable and group, thus leading to the conclusion that normal data distribution can be expected for our data. Mixed ANOVA and figures were provided to show the chronological sequence within the TSST-G for an overall view of stress reactivity. To examine the habituation effects across the three TSST-Gs analyses of variance (ANOVA) were conducted for cortisol, sAA, SDNN, RMSSD, systolic and diastolic pressure, STAI-S, PASA, and MDBF. All requirements were checked before calculation (e.g., normal distribution of residuals, sphericity/homoscedasticity, scaling). If requirements were not fulfilled alternative tests or correction methods (Greenhouse-Geisser for sphericity) were used. First, the ANOVA for repeated measures was used including all participants to show differences between the within-subjects factor time (three points of measurement: t0, t1, t2). All dependant variables were measured separately. Second, the mixed ANOVA for repeated measures was used to analyse the group x time effect of foreign deployment versus control group on the habituation effects to the repeated TSST-G. Therefore, the within-subject factor was time and the between-subject factor was group. Homogeneity of the error variances, as assessed by Levene’s test (*p* > 0.05) was tested as well as homogeneity of covariances, as assessed by Box’s test (*p* > 0.05). Due to the unbalanced proportions of the variable mission abroad in females (seven with and 14 without mission abroad) and the small sample size of women in the experimental group, only male soldiers were considered for the calculations of group x time effects. A χ^2^-test for association was conducted between gender and mission abroad. The expected cell frequencies were less than the minimal required five for females for most biological variables in the experimental group. Therefore, we decided to exclude females from group analyses. For significant results bonferroni-adjusted *post-hoc* tests showed which variables (point of measurement/group) differed from each other. The covariates age and sex were taken into account in all calculations which involved biological variables to examine and control for any statistical influence on the outcome. The total number of missions abroad of a soldier prior to study inclusion was additionally taken into account as a covariate for the group x time effects in men. All reported analyses were tested on the significance level of *p* < 0.05. As a measure for effect size for the ANOVAs the partial eta squared (*η2*) was reported.

## 3 Results

### 3.1 Descriptive data

**FIGURE 2:**
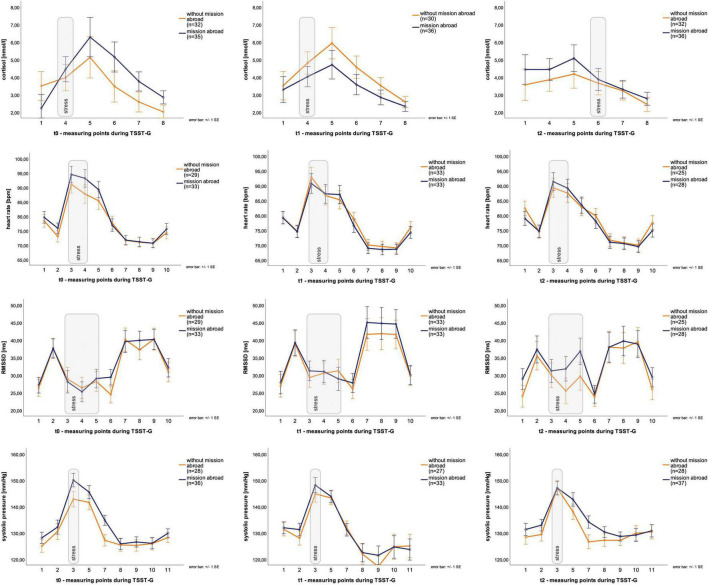
Stress response within Trier Social Stress Test for groups (TSST-G) (t0, t1, t2). Y-axis values: absolute values for cortisol (nmol/l), heart rate (bpm), RMSSD (ms), systolic pressure (mm/Hg). Stress: induced stress exposure following TSST-G protocol; width of stress bars reflects the number of measurement points within the variable; values are means (± SE).

Descriptive data for male deployed and non-deployed soldiers at baseline measure is illustrated in Table 1. The average stay for a mission abroad was about 3 months long (m = 3.23, sd = 1.8). None of the participants had a posttraumatic stress disorder at any measurement time point (t0, t1, t2). Descriptive data representing all included male and female soldiers can be found in the Supplementary Table 1). The gender distribution was 21 (23%) female and 70 (77%) male soldiers. Among females, 14 were in the control group and seven in the experimental group, whereas 37 male soldiers had a mission abroad and 33 did not. The activation of biophysiological variables was tested (absolute values) to show changes in the groups with/without mission abroad within the TSST-G experiments for men. Exemplary results can be found in Figure 2. For cortisol, heart rate, RMSSD and systolic pressure with TSST-G t0, t1, and t2, the mixed ANOVA revealed non-significant results for all group and time x group effects. Most time effects were significant with *p* < 0.001 (Table 2).

**TABLE 1:** Descriptive data for male soldiers with and without mission abroad.

Variable	*N* (cg/eg)	M (cg/eg)	SD (cg/eg)	Range (cg/eg)	*T*-test
Age	33/37	29/34	7.13/7.66	19–56/21–50	*t*(68) = −3.08*
BMI	32/36	24/26	2.81/3.12	19–29/21–35	–
Cortisol (nmol/l)	24/25	4/2	7.42/1.99	<1–30/<1–9	–
Alpha-amlyase (U/ml)	21/16	61/56	42.67/37.85	14–181/11–136	–
Heart rate (bpm)	21/21	78/80	11.72/13.27	54–99/57–101	–
HRV-RMSSD (ms)	21/21	26/27	11.10/15.73	9–53/7–65	–
HRV-SDNN (ms)	21/21	51/47	17.68/22.33	23–95/14–113	–
Diastolic pressure (mm/Hg)	20/32	78/79	11.22/10.16	55–100/46–102	–
Systolic pressure (mm/Hg)	20/32	125/127	13.04/13.80	89–142/83–153	–
STAI-S before	33/37	35.30/35.65	6.53/7.28	24–60/23–54	–
STAI-S after	33/37	39.97/38.76	6.98/8.96	23–55/25–67	–
PASA	32/36	−1.13/−1.19	1.25/1.30	−3.75 to 1.63/−3.63 to 1.88	–
MDBF good/bad mood before	33/36	17.39/17.31	2.02/2.56	13–20/8–20	–
MDBF good/bad mood after	33/37	15.15/15.51	2.80/3.02	9–20/5–20	–
MDBF alertness/tiredness before	33/37	13.18/13.00	2.81/3.76	8–20/5–19	–
MDBF alertness/tiredness after	33/36	12.76/14.03	2.39/3.19	8–18/5–19	–
MDBF calmness/restlessness before	32/37	16.28/16.73	2.13/2.76	10–20/9–20	–
MDBF calmness/restlessness after	33/37	14.70/14.97	3.29/3.40	8–20/6–20	–

*N*, number; *M*, mean; *SD*, standard deviation; cg, control group (no mission abroad); eg, experimental group (mission abroad); *significant at the <0.05 level.

STAI-S, state-trait-anxiety inventory: before and after mental stress task; PASA, primary appraisal secondary appraisal: overall stress index; MDBF, multidimensional mood state questionnaire: before and after mental stress task. Values for cortisol, amylase, systolic pressure, diastolic pressure, heart rate and heart rate variability (HRV) indicate resting values—first measurement after resting phase in the Trier Social Stress Test for groups (TSST-G) (t0).

**TABLE 2:** Results for the group x time interaction effects (mixed ANOVA) in men within Trier Social Stress Test for groups (TSST-Gs).

Variable	Effect	*F*	*df*	*p*	Partial η*^2^*
Cortisol—t0	Time x group	0.83	1.90, 121.53	0.43	–
	Time	1.90	1.90, 121.53	0.16	–
	Group	0.28	1, 64	0.60	–
Cortisol—t1	Time x group	0.63	1.77, 111.34	0.52	–
	Time	1.61	1.77, 111.34	0.21	–
	Group	0.11	1, 63	0.74	–
Cortisol—t2	Time x group	0.75	1.85, 120.18	0.46	–
	Time	1.91	1.85, 120.18	0.16	–
	Group	0.67	1, 65	0.42	–
Heart rate—t0	Time x group	0.93	2.82, 166.45	0.42	–
	Time	3.94	2.82, 166.45	0.01*	0.06
	Group	0.48	1, 59	0.49	–
Heart rate—t1	Time x group	0.34	2.85, 179.23	0.79	–
	Time	5.48	2.85, 179.23	<0.01*	0.08
	Group	<0.01	1,63	0.96	–
Heart rate—t2	Time x group	0.53	3.52, 175.85	0.69	–
	Time	5.03	3.52, 175.85	<0.01*	0.09
	Group	0.02	1, 50	0.89	–
RMSSD—t0	Time x group	0.47	5.85, 345.22	0.83	–
	Time	3.72	5.85, 345.22	<0.01*	0.06
	Group	0.38	1, 59	0.54	–
RMSSD—t1	Time x group	0.64	4.34, 273.63	0.65	–
	Time	4.64	4.34, 273.63	<0.01*	0.07
	Group	0.70	1, 63	0.41	–
RMSSD—t2	Time x group	0.84	4.77, 238.53	0.52	–
	Time	3.81	4.77, 238.53	<0.01*	0.07
	Group	0.90	1, 50	0.35	–
Systolic pressure—t0	Time x group	0.99	6.40, 390.08	0.44	–
	Time	1.29	6.40, 390.08	0.26	–
	Group	0.77	1, 61	0.38	–
Systolic pressure—t1	Time x group	0.70	2.78, 158.19	0.55	–
	Time	4.70	2.78, 158.19	<0.01*	0.08
	Group	0.13	1, 57	0.72	–
Systolic pressure—t2	Time x group	0.79	5.41, 335.45	0.56	–
	Time	2.03	5.41, 335.45	0.07	–
	Group	0.42	1, 62	0.52	–

Group, with/without mission abroad; time, measurement points within one TSST-G; effect sizes are shown if results are significant; cortisol (nmol/l), heart rate (bpm); root mean square of successive differences between normal-to-normal inter-beat intervals (RMSSD) (ms), systolic pressure (mm/Hg).

*Significant at the <0.05 level; df, degrees of freedom.

### 3.2 Psychobiological habituation of stress response

**FIGURE 3:**
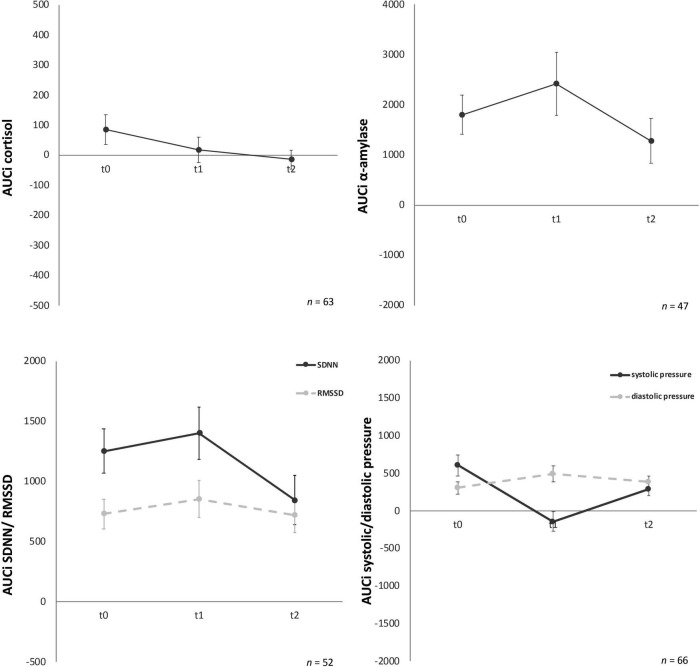
Stress response of cortisol, α-amylase, standard deviation of all normal-to-normal inter-beat intervals (SDNN) and root mean square of successive differences between normal-to-normal inter-beat intervals (RMSSD), systolic and diastolic pressure for the three time points (t0, t1, t2). Y-axis values: AUCi, area under the curve with respect to increase for cortisol (nmol/l), amylase (U/ml), SDNN/RMSSD (ms), systolic/diastolic pressure (mm/Hg). Values are means (± SEM).

Psychobiological habituation effects then were examined across the three TSST-Gs for all participants (female and male). For cortisol AUCg and AUCi no significant decrease between t0, t1, and t2 were given [*F* (1.36, 81.37) = 0.03, *p* = 0.93 and *F* (1.58, 95.03) = 0.36, *p* = 0.65], no habituation effects could be found (Figure 3).

For AUCg amylase no significant differences over time [*F* (2, 88) = 0.06, *p* = 0.94) were found either. Similarly, AUCi amylase (Figure 3) showed no significant effects over time [*F* (2, 88) = 1.43, *p* = 0.25].

For AUCg and AUCi SDNN, again no significant differences for the three time-points were given, *F* (2, 100) = 0.41, *p* = 0.67 and *F* (2, 100) = 0.19, *p* = 0.82, respectively (Figure 3). AUCg and AUCi RMSSD showed no significant effects either [*F* (1.78, 89.02) = 0.33, *p* = 0.70 and *F* (2, 100) = 0.56, *p* = 0.57, respectively).

Analyses of variance for systolic pressure determined that AUCg did not show any significant differences between the three time points, *F* (2, 126) = 2.83, *p* = 0.06. Similarly, AUCi systolic pressure (Figure 2) did not habituate over time either, *F* (2, 126) = 0.60, *p* = 0.55. No significant change over time for the three timepoints were given for AUCg diastolic pressure [*F* (2, 126) = 0.95, *p* = 0.39] and AUCi diastolic pressure [*F* (2, 128) = 1.57, *p* = 0.21] (Figure 3).

**FIGURE 4:**
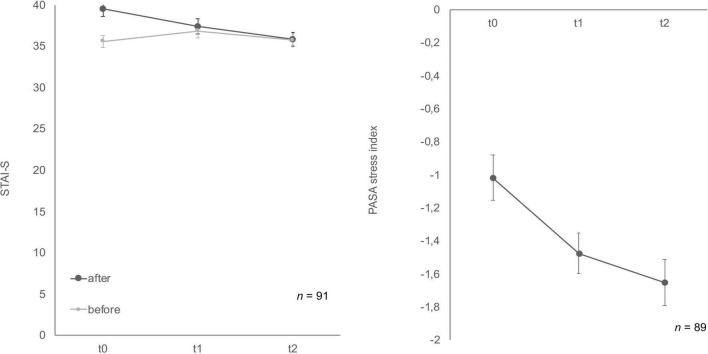
Stress response of state-trait-anxiety inventory: before and after mental stress task (STAI-S) before/after mental stress and primary appraisal secondary appraisal (PASA) during Trier Social Stress Test for groups (TSST-G) for the three measurement points (t0, t1, t2). Values are means (± SEM).

State-trait-anxiety inventory: before and after mental stress task for anxiousness revealed a significant habituation effect after the TSST-Gs for the three time points, *F* (2, 174) = 10.11, *p* < 0.001, partial η^2^ = 0.104 (Figure 4), whereas the anticipatory anxiousness before stress did not show any differences, *F* (2, 180) = 1.75, *p* = 0.18. In the Bonferroni-adjusted post-hoc analysis STAI-S after mental stress showed a significant decrease (*p* = 0.03) in t0 and t1 [2.10, 95%-CI (0.15, 4.06)] and between t0 and t2 [*p* < 0.001, 3.59, 95%-CI (1.58, 5.60)].

Cognitive appraisal also habituated over time (Figure 4). The ANOVA determined that PASA showed a statistically significant difference between measurements, *F* (1.77, 148.46) = 11.65, *p* < 0.001, partial η^2^ = 0.12. Bonferroni-adjusted post-hoc analysis revealed a significant decrease (*p* < 0.01) in t0 and t1 [0.40, 95%-CI (0.12, 0.67)] as well as between t0 and t2 [*p* < 0.001, 0.59, 95%-CI (0.24, 0.95)].

**FIGURE 5:**
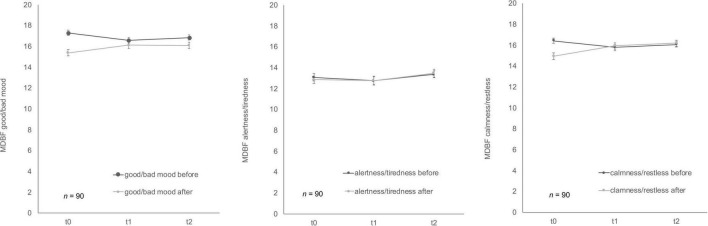
Stress response of multidimensional mood state questionnaire (MDBF) before and after stress exposure during Trier Social Stress Test for groups (TSST-G) for the three measurement points (t0, t1, t2). Values are means (± SEM).

Soldiers’ mood showed differences over time for MDBF scale (Figure 5) good/bad mood before and after mental stress as well as for the dimension calmness/restlessness after the TSST-G. Good/bad mood before indicated habituation effects over time, *F* (2, 178) = 3.41, *p* = 0.04, partial η^2^ = 0.04. Bonferroni-adjusted *post-hoc* analysis revealed a significant decrease (*p* = 0.02) between t0 and t1 [0.69, 95%-CI (0.10, 1.28)]. In comparison, good/bad mood after mental stress increased *F* (2, 170) = 4.94, *p* = 0.01, partial η^2^ = 0.06. Bonferroni-adjusted *post-hoc* analysis showed a significant difference (*p* = 0.02) in t0 and t1 [−0.74, 95%-CI (−1.41, −0.08)] as well as between t0 and t2 [*p* = 0.03, −0.79, 95%-CI (−1.52, −0.06)]. Calmness/restless after mental stress also significantly increased *F* (2, 172) = 11.84, *p* < 0.001, partial η^2^ = 0.12. Bonferroni-adjusted *post-hoc* analysis showed a significant difference (*p* < 0.01) in t0 and t1 [−1.13, 95%-CI (−1.83, −0.43)] as well as between t0 and t2 [*p* < 0.001, −1.38, 95%-CI (−2.12, −0.63)]. For the bipolar dimension alertness/tiredness before no statistically significant differences for the three timepoints were given, *F* (2, 180) = 1.12, *p* = 0.33. Similarly, alertness/tiredness after showed no significant effects either, *F* (2, 168) = 2.24, *p* = 0.11. No habituation effects could be found for the dimension calmness/restless before the mental stress task, *F* (2, 178) = 2.41, *p* = 0.09.

### 3.3 Influence of mission abroad on TSST-G stress response

**FIGURE 6:**
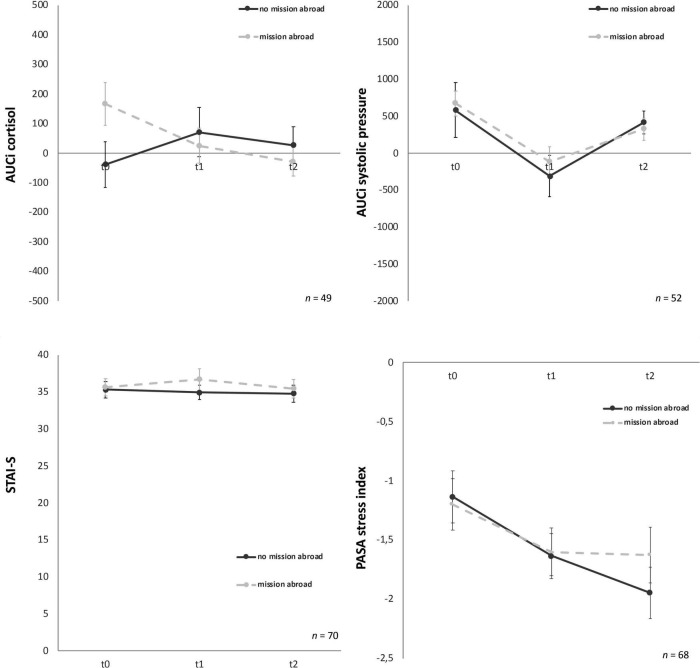
Stress response of cortisol, systolic pressure, state-trait-anxiety inventory: before and after mental stress task (STAI-S) and primary appraisal secondary appraisal (PASA) for the three time points (t0, t1, t2). Y-axis values: AUCi, area under the curve with respect to increase for cortisol (nmol/l) and systolic pressure (mm/Hg). STAI-S before mental stress and PASA during Trier Social Stress Test for groups (TSST-G); values are means (± SEM).

Group effects for male soldiers with and without mission abroad over t0, t1, and t2 were subsequently calculated. The covariates age for biological variables and number of missions abroad for all variables were taken into account. For AUCi cortisol a significant interaction between time and group, *F* (1.61, 72.5) = 4.13, *p* = 0.03, partial η^2^ = 0.08 was found. On closer examination it got clear that the experimental and the control group showed differences as a trend from the start (t0), *F* (1, 47) = 3.88, *p* = 0.06 but adjust over time (Figure 6). Therefore, no significant difference in t1 or t2 could be found.

For all other biological, physiological and psychological parameters no significant time x group interaction effects could be detected (Table 3). Mission abroad did not show any significant influence on the stress response outcome when compared to non-deployed soldiers. Exemplary representations of these results can be found in Figure 6.

**TABLE 3:** Results for the group x time interaction effects (mixed ANOVA) in men across trier social stress test for groups (TSST-Gs) (t0, t1, t2).

Variable	Effect	*F*	*df*	*p*	Partial η^2^
Cortisol (AUCg)	Time x group	0.94	1.44, 64.79	0.37	–
	Time	0.42	1.44, 64.79	0.60	–
	Group	0.09	1, 45	0.76	–
Amylase (AUCg)	Time x group	0.12	2, 66	0.89	–
	Time	0.75	2, 66	0.48	–
	Group	0.22	1, 33	0.64	–
Amylase (AUCi)	Time x group	0.61	1.57, 51.65	0.51	–
	Time	0.45	1.57, 51.65	0.60	–
	Group	0.32	1, 33	0.58	–
SDNN (AUCg)	Time x group	0.46	2, 74	0.63	–
	Time	0.01	2, 74	0.99	–
	Group	<0.01	1,37	0.97	–
SDNN (AUCi)	Time x group	0.51	2, 74	0.60	–
	Time	0.33	2, 74	0.72	–
	Group	1.13	1, 37	0.29	–
RMSSD (AUCg)	Time x group	0.44	2, 74	0.65	–
	Time	0.08	2, 74	0.92	–
	Group	0.11	1, 37	0.75	–
RMSSD (AUCi)	Time x group	0.03	1.59, 58.88	0.95	–
	Time	0.16	1.59, 58.88	0.80	–
	Group	0.62	1, 37	0.44	–
Systolic pressure (AUCg)	Time x group	2.38	2, 94	0.10	–
	Time	4.96	2, 94	<0.01*	0.10
	Group	0.02	1, 47	0.89	–
Systolic pressure (AUCi)	Time x group	0.07	2, 94	0.94	–
	Time	1.75	2, 94	0.18	–
	Group	0.02	1, 47	0.89	–
Diastolic pressure (AUCg)	Time x group	0.98	2, 94	0.38	–
	Time	2.45	2, 94	0.09	–
	Group	<0.01	1,47	0.96	–
Diastolic pressure (AUCi)	Time x group	0.27	2, 94	0.77	–
	Time	0.88	2, 94	0.42	–
	Group	0.11	1, 47	0.75	–
STAI-S before stress	Time x group	0.77	2, 132	0.47	–
	Time	1.06	2, 132	0.35	–
	Group	0.27	1, 66	0.61	–
STAI-S after stress	Time x group	0.89	2, 126	0.41	–
	Time	5.13	2, 126	<0.01*	0.08
	Group	0.05	1, 63	0.83	–
PASA	Time x group	0.70	1.72, 104.85	0.50	–
	Time	5.88	1.72, 104.85	<0.01*	0.09
	Group	0.15	1, 61	0.70	–
MDBF good/bad mood before stress	Time x group	1.09	2, 130	0.34	–
	Time	2.85	2, 130	0.06	–
	Group	0.45	1, 65	0.51	–
MDBF good/bad mood after stress	Time x group	1.49	2, 124	0.23	–
	Time	3.49	2, 124	0.03*	0.05
	Group	0.14	1, 62	0.71	–
MDBF alertness/tiredness before stress	Time x group	0.05	2, 132	0.95	–
	Time	0.67	2, 132	0.52	–
	Group	<0.01	1,66	0.99	–
MDBF alertness/tiredness after stress	Time x group	0.25	2, 120	0.78	–
	Time	1.70	2, 120	0.19	–
	Group	2.81	1, 60	0.10	–
MDBF calmness/restlessness before stress	Time x group	0.90	2, 130	0.41	–
	Time	1.14	2, 130	0.32	–
	Group	0.05	0, 65	0.83	–
MDBF calmness/restlessness after stress	Time x group	0.04	2, 126	0.96	–
	Time	13.08	2, 126	<0.01*	0.17
	Group	0.98	1, 63	0.33	–

Group, with/without mission abroad; time, t0, t1, t2; AUC, area under the curve; effect sizes are shown if results are significant. STAI-S, state-trait-anxiety inventory; PASA, primary appraisal secondary appraisal: overall stress index; MDBF, multidimensional mood state questionnaire. *Significant at the<0.05 level; df, degrees of freedom.

## 4 Discussion

### 4.1 Habituation effects in soldiers and the influence of mission abroad

No habituation effects over the three time-points were detectable for any of the biological and physiological variables cortisol, sAA, RMSSD, SDNN, systolic and diastolic pressure in female and male soldiers. However, psychological stress data indicated habituation effects for PASA and STAI-S after mental stress as well as for MDBF dimension good/bad mood before mental stress. According to the psychological scales, soldiers tend to show less anxiety and stress-appraisal but showed impaired mood. After the mental stress task from the TSST-G the dimensions calmness/restlessness and good/bad mood increased significantly, therefore soldiers tended to be more relaxed and were in a better mood over the three time points.

Foreign deployment in male soldiers did not seem to have any significant effects neither on the physiological or biological nor on the psychological stress response, both groups reacted in the same way to mental stress over time. Only cortisol showed marginally significant differences between the groups before the intervention (t0) which may result from the lack of randomization of the groups due to our study design.

Our findings show a different pattern than findings of other research groups that found habituation mostly for cortisol/HRV and an increase in sAA as an anticipation reaction (21, 27, 51, 52). These contrary results could be explained by using different study designs. Most research on repeated mental stress focused on relatively short time periods between the repetition, mostly a few days or at most weeks (51, 52), whereas our study focused on long-time effects with 3 months between t0 and t1 and even a 1 year period for the t2 follow-up. Therefore, these results reflect the stress adaptation mechanism to a repeated stressor over a relatively longer time period. The biological fight or flight reaction stays stable over time and a reaction is provoked every single time a potential threatening stressor is presented, whereas an adaptation to the stressor can be expected if the stressor is presented more regularly within a shorter period of time (53). This possible explanation is supported by one of the habituation criteria from Thompson and Spencer (22) that implies that rapid frequency (time) of a stressor accelerates and enhances habituation. It could be possible that the HPA and SAM stress activation have a sensitive time frame for “remembering” repeated stress, which leads to already shown habituation or activation changes, whereas when the same stressor repeatedly occurs outside of this time frame no memorizing and changes in the biological stress answer patterns can be found anymore.

The group setting could be another possible impact on the different stress activation when compared to previous studies which had mostly been performed using the single TSST (52, 54, 55). The soldiers were confronted with other participants right from the start and they did not know who the other participants were going to be for each TSST-G. This generated a situation where the participants experienced social evaluation which increased social-self threat, shame and a decrease in self-esteem through the possibility of their performance being judged by others (56). Psychosocial stress situations activate the HPA-axis response (57) and the effect of the group setting is well-established to provoke a robust psychobiological stress response (33). Especially the TSST-G evoked an even higher response to the stressor then in the single setting (31). This could lead to a suppressed biological habituation because of the instability of a changing group for each time-point. These conditions supported the usability of the repeated TSST-G in our case because in this study a stable biological stress response without significant changes occurred throughout the three measurements. The TSST-G seems to be a reliable instrument with the ability to provoke a stress response without biophysiological habituation effects even if used repeatedly on the same population after at least 3 months. Finally, the sample size from our BEST-study was larger than in other studies (36, 52, 58). Smaller sample sizes mostly result from the complex and time-intensive procedure of the single TSST.

The results of the psychological changes of anxiety and stress perception to a mental task measured with PASA and STAI-S are in line with the literature (51, 58, 59). The results in this study showed habituation of anxiousness after the mental stress task and cognitive appraisal for t1 and t2 in comparison to the baseline t0. These findings were expected and result from the already known task and procedure for the second and third stress test. Subjective indices of activation have been shown to decrease over time whereas the performance level even increases, due to the psychological habituation to the stimulus it is possible to concentrate more on the performance level (28). Consequently, this may lead to the conclusion that in this study the biological adaptation program is not as sensitive for delayed stress stimuli as the psychological answer. Briefly, participants remember the past stress situation in terms of mood, anticipation and the attitude towards it and react to the known stimulus differently (51, 58, 59), whereas the body does not change the adaptation program when the repetition of the stress stimuli is delayed (53). This leads to the conclusion that it could be important to consider the differentiation between the biological and psychological stress answer system to mental stress, especially when it comes to coping and buffering mechanisms.

There is a lack of research about changes of the psychobiological stress reaction after foreign deployment in soldiers, especially regarding to a stable and consistent stress response before and after mission abroad. To the best of our knowledge this is the first study showing psycho-physio-biological stress responses to acute mental stress in relation to a mission abroad. Our results show that the reaction and adaptation to repeated stress does not seem to be influenced by a mission abroad and the experiences coming along with it. Different reasons may play a role in this phenomenon. Resilience of the soldiers like family support can act as compensatory mechanisms during deployment experiences (60). It is known that the recovery phase and a supportive environment after stress has a major impact on processing negative experiences and consequently psychological health (61). For a wider perspective, psychosocial aspects after mission abroad which are also collected in the context of the BEST-study could be associated with stress response in a next step. Personality characteristics like hardiness or group cohesion may also function as helpful traits for coping (62). Furthermore, the term “healthy soldier effect” describes a lower mortality rate in military personnel than in the general population due to physical exercise, regular medical examinations and direct access to medical care (63). Nevertheless these findings depend on the type and area of deployment as well as the follow-up time (64). Clearly, more research considering potential moderating variables is mandatory to clarify the underlying mechanisms for the shown resilience.

### 4.2 Limitations

The sample size was limited and unbalanced with respect to the distribution of male and female participants. However, this distribution reflects the unequal gender distribution in the military in general, even if 23 % women in our sample is a higher proportion when compared to the overall distribution in the German army, which was 12.3 % in 2019 (65). Therefore, any generalization to the whole population has to be confirmed in a non-soldier sample to increase extern validity. The sample in this study included healthy, middle-aged German soldiers that undergo regular medical examination. This may also positively affect the extrapolation of the study results to the general population. The overall healthiness of the soldiers could allow the conclusion that the results could hold also for different samples. No randomisation for deployed and non-deployed soldiers was possible as the soldiers were appointed by the German military for a mission abroad which could lead to selection bias from the start. In this context the group x time interaction effect for cortisol could be explained like mentioned above. It must be taken into consideration that the participating soldiers were deployed to rebuilding zones, which cannot be compared with the stress response and habituation changes in soldiers experiencing acute mortal danger and continual front-line status with little to no opportunity for decompression and relaxation. The focus in this study group did not include major traumatic stress, it solely dealt with social stress factors e.g., sleep deprivation or being separated from family. Less than half of the participants went through all three stress tasks which could lead to selection bias towards more conscientious soldiers. Also, the time periods between measurements differed between the individuals because the soldiers did not all go to a mission abroad at the same time. The admission to the second and third stress test could not precisely be planned because the soldiers were allocated all over the country, therefore the 3 months and 1 year time lag differed slightly. Further investigation and a different population are needed to reduce these biases and to confirm the presented results.

To our knowledge this study is one of the first to examine psychobiological habituation effects in a group setting on repeated mental stress over prolonged time periods in soldiers. The method of calculating AUCi and AUCg allowed us to display changes of the biological stress answer during the whole TSST-G session and therefore we were not limited to only one specific time point, which always leads to informational loss. This approach covered for individual differences like a prolonged or delayed stress response. It has to be mentioned that the AUC is not suitable for investigations within one TSST-G trial because it may mask differences at specific time points. The used TSST-G procedures provided a standardized protocol for great comparability and additionally we were able to address the question whether a mission abroad can lead to changed stress adaptation.

## 5 Conclusion

In summary our results suggest that the stress sensitive systems of our body do not habituate or adapt to repeated mental stress over longer time periods while the affective response shows changed experience and behavior patterns. In particular, no effects of foreign deployment on the habituation to mental stress in soldiers could be found which indicates the possible conclusion that the partially stressful experiences during a mission abroad have no direct effect on the psychobiological adaption program. These findings are important for the research to stress-related mental health and disease in military personnel and their resilience. To gain an insight in the potential underlying mechanism would be of great interest, especially for expanding and complementing suitable supportive interventions before and after missions abroad.

## Data availability statement

The raw data supporting the conclusions of this article will be made available by the authors, without undue reservation.

## Ethics statement

The studies involving human participants were reviewed and approved by Ethics Commitee of Ulm University, Ulm, Germany. The patients/participants provided their written informed consent to participate in this study.

## Author contributions

TM, CW, and MR: conceptualization. CW and MR: methodology. TM: formal analysis and writing—original draft preparation. D-SR, MR, and TM: investigation. CW, PR, and MR: writing—review and editing. TM, MR, D-SR, JM, and FP: formal analysis. CW, HG, SB, BF, and H-PB: funding acquisition. All authors have read and agreed to the published version of the manuscript.
