# Supplementary material for: Mental but no bio-physiological long-term habituation to repeated social stress: A study on soldiers and the influence of mission abroad
Source: Front Psychiatry. 2022 Dec 15;13:1011181. doi: 10.3389/fpsyt.2022.1011181 (PMC9797525; doi:10.3389/fpsyt.2022.1011181)
Supplement: Supplementary file 1 [file Table_1.docx]

**Supplementary Table 1.** Descriptive data for female and male soldiers

| Variable | *N* (f/m) | *M* (f/m) | *SD* (f/m) | Range (f/m) | T-test |
| --- | --- | --- | --- | --- | --- |
| Age | 21/70 | 29/32 | 5.75/7.86 | 19-45/19-56 |  |
| BMI | 21/68 | 24/25 | 3.23/3.03 | 18-30/19-35 |  |
| Cortisol [nmol/l] | 14/49 | 3/3 | 3.05/5.39 | <1-11/<1-30 |  |
| Alpha-amlyase [U/ml] | 10/37 | 35/59 | 15.75/40.21 | 12-55/11-180 |  |
| Heart rate [bpm] | 27/26 | 79/82 | 11.47/13.16 | 54-99/57-101 |  |
| HRV-RMSSD [ms] | 11/42 | 19/27 | 7.12/13.45 | 8-31/7-65 |  |
| HRV-SDNN [ms] | 11/42 | 35/79 | 11.83/19.99 | 20-57/14-113 | *t*(51)=-2.10* |
| Diastolic pressure [mm/Hg] | 14/52 | 78/78 | 7.63/10.47 | 66-94/46-102 |  |
| Systolic pressure [mm/Hg] | 14/52 | 119/126 | 9.72/13.43 | 106-134/83-153 |  |
| STAI-S before  STAI-S after | 21/70  21/70 | 35.81/35.49  40.05/39.33 | 6.95/6.89  8.52/8.05 | 24-55/23-60  24-61/23-67 |  |
| PASA | 21/68 | -0.53/-1.17 | 1.33/1.27 | -3.0-1.75/  -3.8-1.9 |  |
| MDBF good/bad mood before  MDBF good/bad mood after  MDBF alertness/tiredness before  MDBF alertness/tiredness after  MDBF calmness/restlessness before  MDBF calmness/restlessness after | 21/69  20/70  21/70  21/69  21/69  21/70 | 17.10/17.35  15.45/15.34  13.00/13.09  11.00/13.42  16.10/16.52  15.29/14.84 | 2.49/2.30  2.78/2.90  3.59/3.33  3.94/2.88  2.32/2.50  2.47/3.30 | 11-20/8-20  9-20/5-20  6-18/5-20  5-18/5-19  11-20/9-20  9-19/6-20 | *t*(88)=-3.08* |

*Note. N* = number; *M* = mean; *SD* = standard deviation; f = female; m = male; ∗ = significant at the <.05 level

STAI-S = State-Trait-Anxiety Inventory: before and after mental stress task; PASA = Primary Appraisal Secondary Appraisal: overall stress index; MDBF = Multidimensional Mood State Questionnaire: before and after mental stress task. Values for cortisol, amylase, systolic pressure, diastolic pressure, heart rate and HRV indicate resting values – first measurement after resting phase in the TSST-G (t0).
